# Impact Evaluation of Seasonal Malaria Chemoprevention under Routine Program Implementation: A Quasi-Experimental Study in Burkina Faso

**DOI:** 10.4269/ajtmh.17-0599

**Published:** 2017-12-18

**Authors:** Thomas Druetz, Nicolas Corneau-Tremblay, Tieba Millogo, Seni Kouanda, Antarou Ly, Abel Bicaba, Slim Haddad

**Affiliations:** 1 Department of Tropical Medicine, School of Public Health and Tropical Medicine, Tulane University, New Orleans, Louisiana;; 2 Department of Economics, Laval University, Quebec City, Canada;; 3 Institut de Recherche en Sciences de la Santé, Ouagadougou, Burkina Faso;; 4 Department of Preventive and Social Medicine, Faculty of Medicine, Laval University, Quebec City, Canada;; 5 Société d’Études et de Recherches en Santé Publique, Ouagadougou, Burkina Faso

## Abstract

Seasonal malaria chemoprevention (SMC) for children < 5 is a strategy that is gaining popularity in West African countries. Although its efficacy to reduce malaria incidence has been demonstrated in trials, the effects of SMC implemented in routine program conditions, outside of experimental contexts, are unknown. In 2014 and 2015, a survey was conducted in 1,311 households located in Kaya District (Burkina Faso) where SMC had been recently introduced. All children < 72 months were tested for malaria and anemia. A pre–post study with control group was designed to measure SMC impact during high transmission season. A difference-in-differences approach was coupled in the analysis with propensity score weighting to control for observable and time-invariant nonobservable confounding factors. SMC reduced the parasitemia point and period prevalence by 3.3 and 24% points, respectively; this translated into protective effects of 51% and 62%. SMC also reduced the likelihood of having moderate to severe anemia by 32%, and history of recent fever by 46%. Self-reported coverage for children at the first cycle was 83%. The SMC program was successfully added to a package of interventions already in place. To our knowledge, with prevalence < 10% during the peak of the transmission season, this is the first time that malaria can be reported as hypo-endemic in a sub-Sahelian setting in Burkina Faso. SMC has great potential, and along with other interventions, it could contribute to approaching the threshold where elimination strategies will be envisioned in Burkina Faso.

## INTRODUCTION

Despite encouraging signs of a recent reduction in transmission,^1,2^ malaria remains one of the most important public health problems in Burkina Faso. Its entire population lives in high-risk transmission areas. The 2015 World Malaria Report indicates that there were 5,428,000 confirmed cases and 17,000 deaths attributable to malaria out of a total population of 17 million, and the latest malaria indicator surveys revealed a parasitemia prevalence (detected by microscopy) of 46% in children 6–59 months.^3^ With 414 malaria cases per 1,000 persons at risk, Burkina Faso has one of the highest incidence rates in the world.^4^ Most of the malaria cases and deaths occur during or directly after the rainy season (approximately from July to October) and children < 5 are at the greatest risk; surveillance data indicate that they represented 51% of all malaria cases and 69% of all deaths attributable to malaria in 2016.^5–9^

Because of high parasite prevalence and the highly seasonal nature of malaria transmission, most of the Burkinabe health districts are located in areas where the World Health Organization (WHO) has recommended seasonal malaria chemoprevention (SMC, formerly known as “intermittent preventive treatment for children”).^10,11^ Officially recommended by the WHO since 2012, SMC involves the administration of a long-acting antimalarial drug regimen that combines therapeutic and prophylactic effects.^12^ The regimen, commonly one single dose of sulfadoxine–pyrimethamine (SP) and three daily doses of amodiaquine (3AQ), is typically administered once per month to the entire population of children < 5 during malaria transmission season. SMC usually consists of three or four cycles of drug administration per year, depending on the length of the rainy season, and is most suitable in specific regions of the Sahel and sub-Sahel.^10,12,13^

Clinical trials and meta-analyses have demonstrated the efficacy of SMC to reduce malaria incidence during the intervention period and parasitemia prevalence at the end of the transmission season and suggest a positive impact on all-cause mortality.^14,15^ Experimental studies have also found evidence of a lower prevalence of moderate to severe anemia, but this reduction was not always significant across trials.^16–19^ Similarly, some trials suggest that SMC is associated with greater weight gain or improvement of malnutrition indices.^16,17,20^ Monthly administration of SP-3AQ was found to be the drug regimen with the highest efficacy,^21–23^ and using community health workers (CHWs) to deliver it is more cost-effective than using facility-based nurses, immunization outreach clinics, or outreach trekking teams.^24,25^

However, there is a lack of evidence on the protective effect of SMC in natural conditions of implementation outside of experimental contexts.^13,26,27^ This type of evaluation is challenging but necessary; indeed, the effectiveness of public health programs often differs from the efficacy measured in trials because of implementation challenges.^28^ In Burkina Faso, where SMC was recently introduced at the national level, a robust, quasi-experimental study was designed to evaluate its impact on malaria-related outcomes (parasitemia prevalence, anemia prevalence, and occurrence of febrile episodes) in preschool-aged children. To the best of our knowledge, this was the first study to evaluate the impacts of an SMC program implemented under routine program implementation. As of 2017, 11 countries have adopted SMC (Burkina Faso, Chad, Gambia, Ghana, Guinea, Guinea Bissau, Mali, Niger, Nigeria, Senegal, and Togo) at different scales of implementation.^29^ The secondary objective was to assess self-reported SMC coverage among the targeted population and to identify the main factors associated with CHW visits and treatment administration during the first cycle of SMC.

## MATERIALS AND METHODS

### SMC in Burkina Faso.

The National Malaria Control Program of Burkina Faso introduced SMC in 2014 in three health districts, then expanded to 17 districts in 2015 and 54 districts in 2016. The national policy builds upon previous trials and pilot projects conducted in the country.^17,23,30,31^ The program is implemented in partnership with nongovernmental and international organizations that financially support the strategy, such as the Malaria Consortium, the World Bank, and United Nations International Children’s Emergency Fund.

The recommended drug regimen for SMC is SP+3AQ. SP was selected because it is still highly effective for parasite clearance in Burkina Faso, where the gene mutations *pfdhfr* and *pfdhps* associated with SP resistance are rare,^23,30,32^ and is different from artemisinin-combination therapies (ACTs), the first-line treatment of malaria. CHWs administer SP and the first dose of AQ, and give the remaining two daily doses of AQ to the caregiver. Four cycles of SMC are conducted each year—once every 4 weeks during high malaria transmission season. SMC usually starts late July, but the exact starting date fluctuates every year depending on several factors, including logistical considerations and pluviometry.

The target population for SMC comprises all children between 3 and 59 months, excluding those with known allergies to sulfamides or AQ, those under cotrimoxazole treatment, and those severely ill or experiencing a presumptive malaria episode. Children 3–11 months receive one 25-mg dose of sulphadoxine, one dose of 12.5 mg of pyrimethamine and three doses of 75 mg of AQ given over the course of three consecutive days. Children 12–59 months received double doses of SP and AQ. The children’s vaccination booklets are consulted to help determine their age.

SMC is administered by CHWs. In teams of two, they go door-to-door to every household. They sometimes organize distribution sessions at gathering venues such as markets, churches, dwellings, mosques, and fields. After explaining SMC strategy (objective, rationale, risks, and benefits, treatment instructions), they administer treatments and refer children with malaria or danger signs to the closest health center. CHWs fill in forms where they indicate the number of treatments administered in every household, and they keep updated stock management sheets. Drugs are supplied through the Ministry of Health to the health centers, who then allocate them to the CHWs. Nurses in health centers are responsible for coordinating CHWs’ work and for collecting SMC forms. Supervisory visits are conducted by nurses and district health authorities. No supply issues were reported during the first cycle of SMC in 2015.

### Study design.

The impacts of SMC were evaluated through a quasi-experimental study, i.e., a pre–post with nonrandomized control group design.^33^ The population study includes all children aged 3–71 months nested in 1,311 households. Observations from 2014 and 2015 (pre- and posttest, respectively) were used for both the children exposed to SMC in 2015 and those who were not (intervention and control groups, respectively). The intervention (the first cycle of SMC) took place from July 31 through August 3, 2015. In 2014 and 2015, data collection started in mid-August and lasted about 3 weeks. No children were exposed to SMC in 2014. The investigators had no control over the implementation of the intervention. In particular, children were not assigned by the research team to the intervention or control group, the latter of which included all children not reached by the intervention at the time of the posttest survey in 2015. The second study component was aimed at measuring self-reported SMC coverage using posttest observations only.

### Study area and population.

The study was carried out in two urban sectors of the city of Kaya (sectors 6 and 7, out of seven sectors) and in 15 villages located within a 20-km radius around the city. These sectors and villages were selected because they are part of the Kaya Health and Demographic Surveillance System (Kaya HDSS), which collaborates with the study. Further information about the Kaya HDSS is available elsewhere.^34^ Kaya is located 100 km northeast of the capital, Ouagadougou. The climate is sub-Sahelian, with a long continuous dry season from November to June.^35^ In 2013, the total rainfall was 781 mm, 81% of which fell in 3 months–July, August, and September. There are six primary health centers in the study area, four rural and two urban, with a catchment population totaling ∼19,077 individuals.

The 2016 health statistics for Kaya district indicate that the test positivity rate for malaria is 77% in health facilities. Based on the number of cases routinely diagnosed, the average number of malaria infections per child per year is 1.1.^9^ Health care for children < 5 has been free of charge in Kaya since 2011, which increased the demand for services provided at health facilities for febrile children.^36^ Approximately 95% of malaria cases in the region are caused by *Plasmodium falciparum*,^1,17,37^ the principal vectors being *Anopheles gambiae*, *Anopheles funestus*, and *Anopheles arabiensis*.^3,8^

Households in the panel were randomly selected from the population living in the study area. They were all surveyed once a year during the peak malaria transmission season, late August or early September.^5,6^ The panel study was designed in 2011 to evaluate the effects of community case management of malaria on treatment-seeking practices and morbidity outcomes in children < 5 years. The panel originally consisted of 3,002 households selected in two health districts (Kaya and Zorgho). After 2013, funding was downsized, procedures and instruments were changed or simplified, and only households located in Kaya district were kept in the panel. The objective was to create a longitudinal research platform for evaluating natural experiments related to child health. More background information on the panel is available elsewhere.^38^ The population of the present study includes the 1,311 households surveyed in 2014–2015 which had at least one child aged 3–71 months. Although the targeted population for SMC comprises children aged 3–59 months, the age cutoff was extended to 71 months because targeting errors for SMC were anticipated for children between 5 and 6 years old. All households agreed to participate in the study.

### Outcomes.

The study has four outcome indicators: 1) point prevalence of parasitemia defined as a positive rapid diagnostic test (RDT) for the detection of parasite *lactate dehydrogenase* (pLDH) malaria antigen; 2) period prevalence of parasitemia, defined as a positive RDT for the *histidine-rich protein II* (HRP2) malaria antigen; 3) prevalence of moderate to severe anemia, defined, according to the WHO, as a hemoglobin concentration under 10 g/dL^39^; and 4) a history of reported fever in the past 14 days. Whereas the pLDH antigen is cleared from the bloodstream within 3 to 5 days after an effective malaria treatment, the HRP2 antigen persists in the blood for 4 to 5 weeks after parasite clearance.^40–43^ The HRP2-based RDT consequently has a low specificity in posttreatment periods and should be considered a measure of period prevalence.^44–46^ Finally, the HRP2 antigen is expressed only by *P. falciparum*, whereas the pLDH antigen is expressed by any species of *Plasmodium*. A study conducted in Burkina Faso revealed that sensitivity was similar for detecting the two antigens by RDTs (99% for pLDH-based RDT and 100% for HRP2-based RDT), unlike their specificity (94% and 71%, respectively).^37^

Two outcome measures were used for self-reported coverage assessment: 1) a household being visited by a CHW during the first cycle of SMC (household level) and 2) a child receiving a course of SP+3AQ (individual level). The information used to assess coverage came from the household survey in the panel.

### Data collection and testing procedures.

Questionnaires were all based on standardized instruments (Malaria Indicator Surveys)^47^ and Demographic and Health Surveys. Children’s characteristics (e.g., sex and age) were recorded as well as self-reported information about exposure to SMC, presence of recent fever episodes, and malaria-related practices (use of bed nets, treatment-seeking behavior in case of fever, etc.). Self-reported exposure to SMC was cross-validated by reviewing the children’s vaccination booklets, if any (only 60% of children had one). The household questionnaire explored the main socioeconomic characteristics and potential confounding variables: household location; size; distance between the household and the CHW; education and occupation of the parents; preventive practices and knowledge regarding malaria; possessions of the household; and finally water, sanitation, and hygienic practices of the household. Based on this self-reported information and direct observation of the households’ immediate environment (presence of stagnant water, yard cleanliness, and presence of animals in the courtyard), a socioeconomic status index and a hygiene index were created, as described elsewhere.^48^

Data collection was performed by teams of two: one field assistant and one health technician. Surveys were administered by trained field assistants using tablets. All surveyed households and CHW residences were located with a Garmin^®^ GPS device (Olathe, KS).

After completing the questionnaire, all children (excluding neonates) were tested for malaria and anemia. A RDT (RDT SD Bioline AG Pf/Pan^®^, Suwon City, South Korea) was conducted using whole blood via finger prick to detect parasitemia. Results were read and interpreted according to the instructions provided by the manufacturer. If invalid, the RDT was performed a second time. Hemoglobin was measured using a photometer (Hemocue 301^®^, Angelholm, Sweden) and axillary temperature was recorded using a digital thermometer. Test results were entered into a data capture system and into record books as backup. If an eligible child was temporarily absent at the time of the survey, the fieldworkers went back to the household for a second and last time on the following day(s) to complete the tests. In the presence of a positive RDT, anemia and/or signs of malnutrition, the child was referred to the nearest health facility, and a referral note intended to the nurse was given to the parent—healthcare for children has been free of charge in Kaya District since 2011. Only one test was performed per child per year.

### Statistical analyses.

Three different types of analyses were performed: 1) coverage assessment, 2) impact evaluation, and 3) a placebo test as a sensitivity analysis for impact evaluation. All analyses were conducted using the STATA^®^ version 14 (STATA Corp., College Station, TX).

#### Self-reported coverage assessment.

SMC coverage was calculated among the study population by area type: urban, peri-urban, and rural. A multiple logistic model was fitted for the two binary outcomes and associations between these two outcomes, and the set of potential confounding variables (previously described) were investigated. Only postintervention data were used. For the first outcome (receiving the visit of a CHW), the level of analysis was the household, whereas for the second (being administered a course of SP+3AQ), it was the child. In the latter case, a random intercept for households was included in the model to account for the hierarchical structure of the data—children are nested in households. Multicollinearity was ruled out by using the Collin package (STATA Corp.) and verifying that variance inflation factors did not exceed 4. The statistical significance of the Wald χ^2^ test was checked to assess goodness-of-fit. To ensure consistency between the analyses and comparability of the results, the same set of covariates was included in the different models, whatever the outcome or the type of analysis (impact evaluation, sensitivity analysis, and coverage assessment). Therefore, models are not as parsimonious as they could be, but their interpretation is more straightforward and coherent.

#### Impact evaluation.

Two approaches based on the Rubin–Neyman potential outcome framework were used to evaluate the impacts of SMC.^49^ First, the reduction in prevalence of morbidity attributable to the intervention was estimated. The causal effects of the intervention on the population under treatment (average treatment effect on the treated) was computed using a difference-in-differences approach.^50^ This approach is based on the comparison of change (pre- versus postintervention, i.e., 2014 versus 2015) in outcome between the intervention and the control group and has the advantage of controlling for time-invariant confounders.^51,52^ The second approach consisted of calculating the protective effects of the intervention for individuals. The protective effect of SMC is expressed by the regression coefficient for exposure, i.e., the adjusted risk ratio of having a positive outcome in the intervention group versus in the control group.

Average treatment effects on the treated and adjusted risk ratios are derived from the same generalized linear (Poisson distribution) model that was replicated for the four malaria-related outcomes. The Poisson regression with robust variance estimators was preferred over the logistic distribution to obtain model-based risk ratio estimates, as recommended in the literature on impact assessment.^53–55^

The causal models were fitted with a propensity score weighting to control for possible selection bias on the set of potential confounding variables (previously described).^56–58^ To produce the propensity scores, a multiple logistic model was fitted to estimate the adjusted probabilities of selection into the treatment and control groups, which were then used to generate scores to be included in the causal models.^58^ All variables potentially related to the outcome were included in the propensity score, regardless of whether they were related to exposure.^59,60^ The balance of covariates was checked by testing the weighted differences in the means of every covariate between the groups.^58^ No difference was found to be statistically significant at a threshold of 10%.

Random intercepts for children were included to account for the hierarchical structure of the data (observations are nested in children since the unit of analysis is the child × year). Multicollinearity was ruled out using the same procedures as described previously. Goodness-of-fit was assessed by computing the Pearson-based dispersion statistics; they suggested the absence of residuals’ over-dispersion and indicated that the data fitted reasonably well the models. The Akaike information criterion and the Bayesian information criterion were considered when comparing models with and without propensity score weighting.

#### Sensitivity analysis.

Difference-in-differences approaches assume a common trend between the treatment and control group (that is, no time-varying differences in outcomes between groups). To assess the robustness of this assumption, we conducted an antitest (or placebo test) verifying whether variations in outcomes were equivalent between the two groups during 2 years of pre-intervention (2013 and 2014).^61,62^ Material and procedures for data collection in the panel were identical between 2013 and 2015.

### Ethics.

Ethical approval was obtained from the research ethics committee of the University of Montreal Hospital Research Center in Canada and Burkina Faso’s health research committee. Data were used in conformity with Kaya Health and Demographic Surveillance System policy (authorization 1KH2016-02). Written informed consent was obtained every year from every respondent and from the caretakers of children on their behalf. Children with danger signs were immediately referred to a health center.

## RESULTS

### Population characteristics.

In 2015, there were 2,523 children aged 3–71 months in the panel. Among these, 91% (*N* = 2,291) were present at the time of the survey: 1,820 (79%) had received an SMC treatment (intervention group), 462 (20%) had not received an SMC treatment (control group), and 9 (0.4%) did not remember or refused to answer. Population characteristics are summarized in Table 1. Exposure status was not statistically significantly associated (χ^2^ < 0.01; *P* value = 0.947) with RDT results the previous year (2014), indicating no selection issues on the likelihood to receive SMC based on the outcome. Differences were found in certain characteristics between children who received SMC in 2015 and those who did not. As expected, more ineligible children (aged 60–71 months) were found in the control group than in the intervention group (χ^2^ = 70.77, *P* value < 0.001). Children in the intervention group were more likely to live in polygamous households than their counterparts in the control group (χ^2^ = 5.81, *P* value = 0.016). Finally, the mean hygiene score was higher in the intervention group than in the control group (2.42 versus 2.27, *P* value = 0.009).

**Table 1:** Population characteristics

	SMC	No SMC	*P* value
Number of children	1,820	462	
Female	49%	46%	0.343
Age category (3–11, 12–59, and 60–71 months)	10%/75%/15%	10%/57%/32%	< 0.001
Sleep under a bed net	96%	94%	0.077
Parasite *lactate dehydrogenase*-based RDT positive in 2014	90%	90%	0.947
Area (urban/peri-urban/rural)	36%/15%/49%	34%/12%/53%	0.166
Mother is literate	7%	8%	0.692
Head of household is a farmer	77%	79%	0.439
Household is polygamous	42%	36%	0.016
Household owns cattle	39%	41%	0.539
Mean number of siblings < 10 years (SD)	3.7 (1.92)	3.9 (1.92)	0.148
Mean distance to the CHW (SD)	1,261 (1,468)	1,356 (1,262)	0.205
Mean socioeconomic score (from 1 to 4) (SD)	2.64 (1.09)	2.65 (1.09)	0.781
Mean hygiene score (from 1 to 4) (SD)	2.42 (1.12)	2.27 (1.11)	0.009

CHW = community health worker; RDT = rapid diagnostic test; SD = standard deviation; SMC = seasonal malaria chemoprevention. Heterogeneity tests performed: Pearson χ^2^ or analysis of variance.

### Self-reported SMC coverage.

**Figure 1.**
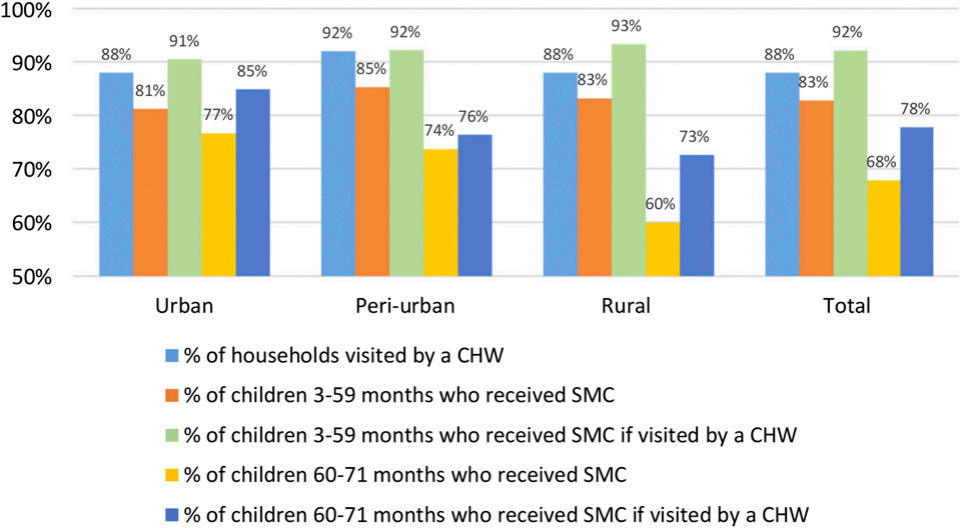
Measures of self-reported coverage for the first cycle of the 2015 SMC round, according the area (urban, peri-urban, and rural). Indicators of self-reported coverage are (from the left to the right) the proportion of households who received the visit of a community health worker (CHW), the proportion of eligible children (3–59 months) who received an SMC treatment, the proportion of eligible children who received an SMC treatment in households visited by a CHW, the proportion of noneligible children (60–71 months) who received an SMC treatment, and the proportion of noneligible children who received an SMC treatment in households visited by a CHW. Chi square tests of homogeneity of variance between the different areas for each of the five indicators are respectively 4.21 (*P* value = 0.122), 2.40 (*P* value = 0.300), 1.40 (*P* value = 0.497), 12.45 (*P* value = 0.002), and 6.88 (*P* value = 0.032). This figure appears in color at www.ajtmh.org.

Figure 1 shows the five different indicators used to assess self-reported coverage. Coverage for households (i.e., receiving a CHW visit during the first cycle of SMC) was 88% in rural areas, 88% in urban areas, and 92% in peri-urban areas. SMC treatment coverage for eligible children aged 3–59 months was greater than 80% in all areas, whereas coverage for children aged 3–59 months living in households reportedly visited by a CHW was greater than 90% in all areas. For every indicator, results from tests of homogeneity of variance between the areas are displayed in Figure 1. They reveal that children too old to be eligible for SMC (i.e., children aged 60–71 months) were more likely to receive SMC treatment in urban than in rural areas (odds ratio [OR] = 2.2, 95% confidence interval [CI] = [1.38–3.47]).

Table 2 reports the associations obtained in logistic regression models between potential covariates and two outcomes of SMC coverage: being visited by a CHW (coverage for households) and receiving an SMC treatment (coverage for children). Coverage for households is not associated with any of the covariates. When compared with those aged 3–11 months, children aged 12–59 months have higher coverage (adjusted OR [aOR] = 2.09, 95% CI = [1.03–4.22]), whereas children aged 60–71 months have lower coverage (aOR = 0.2, 95% CI = [0.09–0.43]). Coverage for children also increases with the hygiene score of the household (aOR = 1.96, 95% CI = [1.35–2.86]).

**Table 2:** Factors associated with SMC coverage at the household- and at the child level (2015, first cycle)

	Coverage for households (being visited by a CHW)			Coverage for children (receiving SMC treatment)		
Household level	Crude OR	aOR [95% CI]	*P* value	Crude OR	aOR [95% CI]	*P* value
Area (ref. urban)						
Peri-urban	1.33	1.38 [0.87–2.19]	0.171	1.20	2.28 [0.71–7.29]	0.166
Rural	0.87	1.00 [0.69–1.45]	0.992	0.91	1.65 [0.75–3.63]	0.216
Literacy of the spouse/mother	1.05	0.98 [0.59–1.63]	0.928	0.96	0.80 [0.30–2.16]	0.658
Farmer (head of the household)	1.02	1.14 [0.78–1.66]	0.494	0.75	1.53 [0.72–3.28]	0.273
Polygamy	0.88	0.91 [0.66–1.25]	0.563	0.75	0.76 [0.38–1.51]	0.432
Cattle possession	0.93	1.14 [0.65–1.18]	0.392	1.01	1.15 [0.57–2.33]	0.696
Number of children < 10 years	0.97	0.99 [0.90–1.08]	0.763	0.94	0.85 [0.71–1.02]	0.077
Distance to the CHW	0.90	0.92 [0.83–1.01]	0.094	0.96	0.85 [0.69–1.05]	0.125
Wealth score	0.95	0.98 [0.85–1.13]	0.805	1.00	0.88 [0.63–1.21]	0.432
Hygiene score	1.10	1.11 [0.97–1.29]	0.139	1.12	1.96 [1.35–2.86]	< 0.001
Individual level						
Female	–	–	–	1.14	1.35 [0.89–2.05]	0.164
Age (ref. 3–11 months)						
12–59 months	–	–	–	1.34	2.09 [1.03–4.22]	0.040
60–71 months	–	–	–	0.48	0.20 [0.09–0.43]	< 0.001
Slept under a bed net	–	–	–	0.66	0.33 [0.10–1.07]	0.064

(a) OR = (adjusted) odds ratio; CHW = community health worker; CI = confidence interval; SMC = seasonal malaria chemoprevention.

### Average treatment effects on the treated.

Estimates derived from regression models with and without propensity score weighting are displayed in Table 3. Results from the weighted regression models show that SMC is associated with a 3.3% reduction (95% CI = [−0.08 to 0.01]) of the estimated point prevalence of malaria and a 24.6% reduction (95% CI = [−0.35 to −0.14]) in the estimated period prevalence of malaria. SMC is also associated with a 16.1% reduction (95% CI = [−0.28 to −0.04]) in the estimated prevalence of moderate to severe anemia, and a 10.2% reduction (95% CI = [−0.19 to −0.01]) in the occurrence of recent fever in the population. These absolute changes in prevalence for malaria-related outcomes are all in the anticipated direction and statistically significant at a threshold of 5% in both weighted and unweighted models (see all 95% CI and *P* values in Table 3). The only exception is the effect on the estimated point prevalence, which is only statistically significant in the unweighted regression model (prevalence difference = −3.4%, 95% CI = [−0.07 to −0.01]). Adding an interaction term between the area (urban/peri-urban/rural) and the exposure status did not change goodness-of-fit of the model or the effect estimates, and the test of likelihood ratio was not statistically significant (*P* = 0.145).

**Table 3:** Impact of seasonal malaria chemoprevention on the prevalence of malaria-related outcomes

	Model without PSW			Model with PSW		
Outcome	Prevalence difference (%)	95% CI	*P* value	Prevalence difference (%)	95% CI	*P* value
Malaria prevalence (1)	−3.4	[−0.07 to −0.01]	0.034	−3.3	[−0.08 to 0.01]	0.129
Parasite *lactate dehydrogenase*-based test						
Malaria prevalence (2)	−24.2	[−0.31 to −0.18]	< 0.001	−24.6	[−0.35 to −0.14]	< 0.001
*Histidine-rich protein II*-based test						
Moderate/severe anemia	−9.7	[−0.18 to −0.02]	0.021	−16.1	[−0.28 to −0.04]	0.009
Recent episode of fever	−15.2	[−0.23 to −0.07]	< 0.001	−10.2	[−0.19 to −0.01]	0.024

CI = confidence interval; PSW = propensity score weighting. Estimates of average treatment effects on the treated are based on a multilevel mixed-effects generalized model without/with PSW. Prevalence difference estimators use a difference-in-differences approach (exposed vs. nonexposed; 2015 vs. 2014).

**Figure 2.**
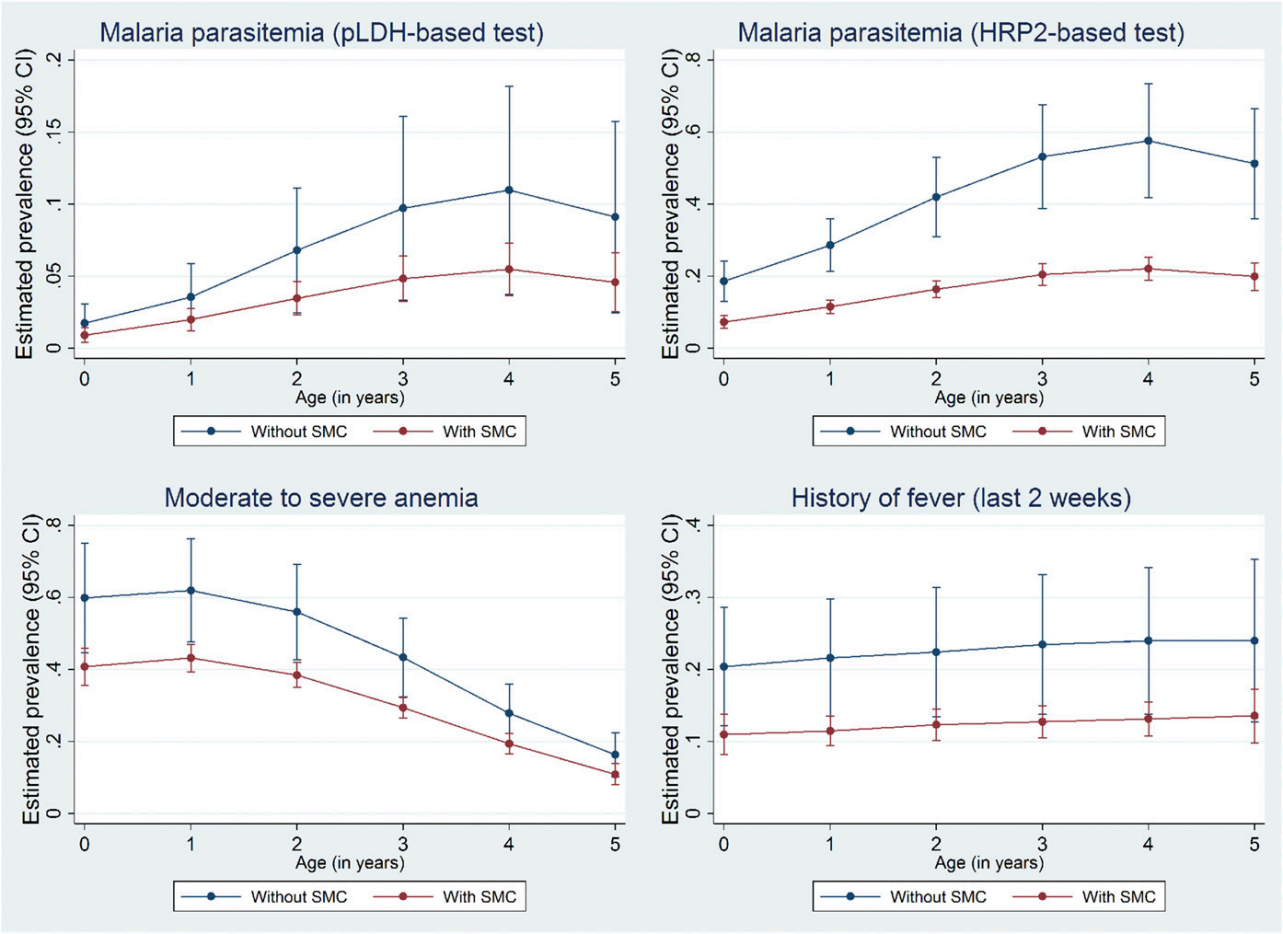
Adjusted prevalence of malaria-related outcomes after the first cycle of the 2015 SMC round, according the age and exposure status of the child. Prevalence estimates were obtained using a multilevel mixed-effects generalized model with propensity score weighting. This figure appears in color at www.ajtmh.org.

For each of the four malaria-related outcomes, Figure 2 displays the estimated prevalence according to exposure status and age of the child in years. For each of the outcomes, the reduction in prevalence reached statistical significance (at a threshold of 5%) in every age category except again for the estimated reduction in point prevalence of malaria in the weighted model (Supplemental File 1).

### Protective effect of SMC.

The protective effect of SMC for individuals is expressed by the risk ratio of having a positive outcome in the intervention group versus in the control group. At baseline, the likelihood of having a positive pLDH-based RDT in children who received SMC the next year was not different than in children who did not receive SMC in 2015 (RR = 1.15, 95% CI = [0.80–1.65], see Supplemental File 2).

The protective effect of SMC against a positive malaria test result via pLDH- and HRP2-based RDT was 51% (RR = 0.49, 95% CI = [0.24–0.99]) and 62% (RR = 0.38, 95% CI = [0.29–0.52]), respectively. SMC also reduced the likelihood of having moderate to severe anemia and recent history of fever by 32% (RR = 0.68, 95% CI = [0.53–0.87]) and by 46% (RR = 0.54, 95% CI = [0.36–0.83]), respectively. All these estimated protective effects are in the anticipated direction (Table 4).

**Table 4:** Protective effects of seasonal malaria chemoprevention

			Model without PSW		Model with PSW	
	Crude RR	*P* value	aRR [95% CI]	*P* value	aRR [95% CI]	*P* value
Malaria prevalence (1)	0.25	0.000	0.51 [0.31–0.84]	0.008	0.49 [0.24–0.99]	0.050
Parasite *lactate dehydrogenase*-based test						
Malaria prevalence (2)	0.36	0.000	0.41 [0.34–0.50]	< 0.001	0.38 [0.29–0.52]	< 0.001
*Histidine-rich protein II*-based test						
Moderate/severe anemia	0.84	0.002	0.76 [0.61–0.93]	0.009	0.68 [0.53–0.87]	0.002
Recent episode of fever	0.52	0.000	0.45 [0.33–0.61]	< 0.001	0.54 [0.36–0.83]	0.004

(a) OR = (adjusted) odds ratio; CI = confidence interval; PSW = propensity score weighting. Estimates are based on a multilevel mixed-effects generalized model without/with PSW.

### Placebo test.

There was no statistical difference in changes between intervention and control groups in pre-intervention years (2013–2014) for any of the outcomes (Supplemental File 3). Therefore, there is no indication that the assumption of similar trends between the two groups is not plausible.

## DISCUSSION

It has been estimated that ∼20,000 deaths and 5 million cases of malaria could be averted with SMC in the Sahel and sub-Sahel regions, if this intervention were widely deployed.^12^ However, the extent of the potential impact is contingent on the coverage and effectiveness that nationwide programs could achieve. This is the first study to evaluate the impact and coverage of a large-scale SMC program under routine program implementation. SMC had immediate and significant protective effects for the four malaria-related outcomes. It significantly reduced the prevalence of malaria and anemia among the population, as well as the occurrence of fever episodes. The robustness of the design, and the congruency of the results over the different outcomes and analytical approaches, substantiate our assertion that SMC had a significant (clinically and statistically) positive impact on child morbidity in the study area. The point prevalence of malaria was reduced by 3.3% points (95% CI = [−0.08 to 0.01]), which translated into a protective effect (adjusted risk ratio) of 51% (95% CI = [0.24–0.99]). The protective effect on the period prevalence was larger, 62% (95% CI = [0.29–0.52]). This is unexpected because HRP2-based RDTs take more time to turn negative after parasite clearance than pLDH-based RDTs, and therefore are less sensitive to measure SMC therapeutic effect between two cycles.^44,45^ A plausible explanation is that a floor effect occurred, as the overall point prevalence of malaria was already low in the study area. This may have contributed to making the evaluation of impacts less precise and reducing the power of statistical tests, as shown by the large 95% CI = for this particular outcome.

The protective effects on malaria infection observed here are lower than the 85% efficacy reported in the only trial that used the same regimen and measured prevalence during the SMC round rather than after.^16^ This was anticipated and is understandable given the study context. Indeed, impacts were assessed in our study under routine program conditions. Taking that into consideration, results are of critical importance for public health planning; decision makers can anticipate that SMC reduces malaria prevalence by at least 50% in a targeted population.

SMC reduced the prevalence of moderate to severe anemia by 16% points (average treatment effect on the treated). This corroborates previous experiments showing that malaria chemoprevention or intermittent treatment is most likely to reduce anemia in rural areas.^17,63^ The protective effect for this outcome was 32%. Similar to results observed from trials, the treatment effect for anemia was lower than that for malaria, which is understandable as only a fraction of the risk of anemia is attributable to malaria.^16,17^ That being said, reducing the risk of anemia by a third through an SMC campaign is a tremendous gain in terms of public health, knowing the heavy burden caused by this condition in children under age 5.^19,64^

Models with propensity score weighting have the potential to better adjust for covariates and, in this analysis, presented signs of better goodness-of-fit (lower Akaike and Bayesian information criteria and smaller dispersion statistics) but at the expense of larger standard errors for the estimates.^56^ Results from both types of models were presented for the reader to appreciate and compare them. Estimates with and without propensity scores are similar for the malaria parasitemia outcomes, suggesting that selection bias is unlikely.^65^ However, they differ moderately for anemia and history of fever. Imbalances between the two groups, either random or systematic, seem to have more influence for these two outcomes. One hypothesis is that, because of a more complex etiology, the association between exposure and these outcomes is more affected by covariates than the association between SMC and malaria parasitemia outcomes.

Local contingencies may have reduced the fidelity of SMC during the first year of its implementation and, consequently, limited its effectiveness in the study area. Self-reported coverage for the first cycle of the 2015 round was 76% and 83% at the household and individual levels (children < 5 years), respectively. Patouillard et al.^24^ used the same delivery method for SMC and found that coverage for children was 100% at the first cycle, then gradually decreased to 69% at the fourth cycle. If a similar drop in coverage was to occur in the study area, the proportion of children fully covered at the end of the round would be 57%. Several factors could explain the limited coverage for households and for children, notably their absence during the rainy season. Indeed, it is common for some household members to spend all day in the fields. Some even leave their homes and temporarily live in shelters closer to the fields.^66^ Also, it is common for young children to stay with their mother during the day and accompany her wherever she goes. This could explain why, even if a household was visited, some children did not receive SMC treatment. Acceptability was likely not an issue because no parent reported having refused SMC for their child. However, troubles with drug administration (difficulties in crushing the tablets into powder and the presence of side effects) could have affected adherence.

None of the observable variables influenced the heterogeneity in coverage among households, and only two variables were associated with the likelihood of receiving SMC: the child’s age and the hygiene score of the household. The first association was expected, if only because age is an eligibility criteria for SMC and because older children are less often at home. The second association with hygiene score suggests that children are more prone to receive SMC if they live in a household whose immediate surroundings are cleaner (courtyard without garbage, animal excreta, stagnant water, etc.), even after adjusting for the area and the household socioeconomic status.^48^ At the same time, results show that households are not more prone to be visited by a CHW if they have a higher hygiene score, indicating no selection bias from the CHW based on the households’ hygiene score. Although it has been demonstrated that domestic environmental measures have a direct preventive impact on malaria,^67^ further investigation is required to interpret the positive association between a household hygiene score and the uptake of an intervention against malaria such as SMC.

The repeated annual surveys also indicate that malaria transmission in the study area is now moderate. Even among children who did not receive SMC, malaria point prevalence was only 6.5%, whereas previous surveys in the panel reported a prevalence of 18.6% (2013) and 15.0% (2014). The overall reduction in malaria prevalence in 2015 might be partly due to the fact that a package of interventions against malaria has been introduced since 2010 in the district, including adoption of ACTs as first-line treatments, community case management, repeated campaigns of universal bed net distribution, community sensitization, and removal of user fees at health centers.^36,38,68–70^ Although better integration of these efforts has been recommended,^71^ SMC contributed positively to this package.

To our knowledge, this is the first time that malaria has been reported as hypo-endemic in a sub-Sahelian setting in Burkina Faso.^72,73^ With all these interventions targeting children < 5, the age pattern of the malaria burden may shift to school-aged children, as shown in other studies.^74,75^ The extension of SMC to school-aged children may be an option to further reduce malaria transmission and approach the threshold where elimination strategies will be envisioned.^76,77^

### Strengths and limits.

Several measures were taken to increase the internal validity of the outcome evaluation in this quasi-experimental study. First, the study was built on a robust design (pre–post with control group), and the conclusions were fueled by four outcome indicators, covering various components of the chain of effects (theoretical replicability).^78–80^ Second, diverse analytical methods were combined to control for observable and time-invariant nonobservable confounding factors: multiple models, propensity score weighting, and difference-in-differences approach. Third, analyses were intentionally conservative; bilateral statistical tests were used, and children were included in the analysis even if they were recently sick and/or received an ACT treatment. Finally, post hoc analyses were conducted to test the hypothesis of similar trends during pre-intervention period and strengthen the robustness of difference-in-differences estimates.

The sensitivity analyses and placebo tests (antitests) do not suggest the presence of selection biases or residual confounding. Coverage assessment has not indicated self-selection issues in treatment administration. It was not possible to assess the impact depending on the adherence level, because implausibly high values were self-reported, as seen in other studies.^81,82^ However, preliminary results from a qualitative study on SMC implementation show a good fidelity to the plan and acceptability of the population, which is congruent with the positive effects on morbidity measured here. The possibility of a selection bias affecting the results is small, although the risk cannot be completely ignored in any quasi-experimental study, no matter how robust.^33^

Finally, the power of the study has been limited by an unexpectedly low malaria transmission rate in the study area. Despite this, significant clinical and statistical gains were observed. The high level of replicability in this study confirms our appreciation of the internal validity and capacity to attribute the gains to SMC. The external validity is limited by the fact that the study took place in only one district. Variations in the implementation of the strategy between districts in Burkina Faso, and between Sahelian countries, are possible. It seems important to replicate this study elsewhere and measure how sensitive the results are to variations in context and in implementation fidelity. In theory, repeated cycles of SMC could contribute to reduce overall malaria transmission.^77^ It was impossible to investigate this hypothesis here because only the postintervention measure was taken a few weeks after the first SMC cycle.

## CONCLUSION

During the first year of its introduction under routine program conditions, SMC already had large and positive impacts for the targeted population. The prevalence of all malaria-related outcomes under study (malaria parasitemia, moderate to severe anemia, and occurrence of febrile episodes) decreased immediately after the first cycle of the SMC campaign. This translated into large and significant protective effects for the children who received the chemoprevention treatment. SMC was successfully added to the package of interventions already in place in the health district where the study was conducted, even if some implementation barriers, notably limited coverage, likely reduced its effectiveness. To our knowledge, it is the first time that malaria can be described as hypo-endemic in the area, with a parasitemia prevalence < 10% during the peak of its transmission season. SMC is an intervention with great potential in Burkina Faso, and along with other interventions, it could contribute to approaching the threshold where elimination strategies will be envisioned.

## Supplementary Material

Supplemental Files.
