# Supplementary material for: Impact Evaluation of Seasonal Malaria Chemoprevention under Routine Program Implementation: A Quasi-Experimental Study in Burkina Faso
Source: Am J Trop Med Hyg. 2017 Dec 18;98(2):524–33. doi: 10.4269/ajtmh.17-0599 (PMC5929206; doi:10.4269/ajtmh.17-0599)
Supplement: Supplementary file 1 [file tpmd170599.SD1.pdf]

SUPPLEMENTAL FILE 1: POPULATION IMPACT OF SEASONAL MALARIA CHEMOPREVENTION ACCORDING AGE OF THE CHILDREN (WEIGHTED MODEL)

| Outcome | Age (in years) | Effect estimated by DiD (%) | Standard error | *z* | *P* > *z* | [95% CI] | |
|---|---|---|---|---|---|---|---|
| Point prevalence of malaria | 0 | −0.9 | 0.006 | −1.45 | 0.146 | −0.021 | 0.003 |
| (Parasite *lactate dehydrogenase*-based RDT) | 1 | −1.9 | 0.012 | −1.52 | 0.129 | −0.043 | 0.005 |
| | 2 | −3.5 | 0.023 | −1.52 | 0.128 | −0.079 | 0.010 |
| | 3 | −4.9 | 0.033 | −1.5 | 0.132 | −0.113 | 0.015 |
| | 4 | −5.6 | 0.037 | −1.5 | 0.135 | −0.129 | 0.017 |
| | 5 | −4.6 | 0.032 | −1.45 | 0.146 | −0.109 | 0.016 |
| Period prevalence of malaria | 0 | −11.4 | 0.026 | −4.46 | 0.000 | −0.164 | −0.064 |
| (*Histidine-rich protein II*-based RDT) | 1 | −17.7 | 0.038 | −4.69 | 0.000 | −0.251 | −0.103 |
| | 2 | −25.7 | 0.056 | −4.59 | 0.000 | −0.367 | −0.148 |
| | 3 | −32.5 | 0.072 | −4.49 | 0.000 | −0.466 | −0.183 |
| | 4 | −35.1 | 0.079 | −4.45 | 0.000 | −0.506 | −0.197 |
| | 5 | −31.4 | 0.073 | −4.31 | 0.000 | −0.456 | −0.171 |
| Prevalence of anemia | 0 | −19.1 | 0.074 | −2.6 | 0.009 | −0.336 | −0.047 |
| | 1 | −19.9 | 0.076 | −2.63 | 0.009 | −0.348 | −0.051 |
| | 2 | −17.9 | 0.069 | −2.61 | 0.009 | −0.314 | −0.045 |
| | 3 | −13.8 | 0.054 | −2.57 | 0.010 | −0.244 | −0.033 |
| | 4 | −9.0 | 0.036 | −2.48 | 0.013 | −0.160 | −0.019 |
| | 5 | −5.2 | 0.022 | −2.33 | 0.020 | −0.096 | −0.008 |
| History of fever | 0 | −9.3 | 0.040 | −2.3 | 0.021 | −0.172 | −0.014 |
| | 1 | −9.8 | 0.043 | −2.3 | 0.021 | −0.182 | −0.015 |
| | 2 | −10.3 | 0.046 | −2.24 | 0.025 | −0.192 | −0.013 |
| | 3 | −10.7 | 0.049 | −2.21 | 0.027 | −0.202 | −0.012 |
| | 4 | −11.0 | 0.050 | −2.18 | 0.029 | −0.208 | −0.011 |
| | 5 | −11.1 | 0.052 | −2.12 | 0.034 | −0.213 | −0.009 |

CI = confidence interval; DiD = difference-in-differences; RDT = rapid diagnostic test.

SUPPLEMENTAL FILE 2: RESULTS FROM THE MEGLM MODEL WITH PROPENSITY SCORE WEIGHTING

Outcome variable (rdt) equals 1 if parasite *lactate dehydrogenase*-based rapid diagnostic test (RDT) is positive.
Exposition variable (smc) equals 1 if the child received seasonal malaria chemoprevention (SMC).
The year (year) of reference is 2014.
The group variable (group) equals 1 if the child belongs to the group that will receive SMC in 2015.

| rdt | RR | Robust<br>Standard error | *z* | *P* > \|*z*\| | [95% CI] | |
|---|---|---|---|---|---|---|
| smc | 0.4939982 | 0.177518 | −1.96 | 0.050 | 0.2442567 | 0.999089 |
| group | 1.146812 | 0.2114906 | 0.74 | 0.458 | 0.7989448 | 1.646143 |
| year | | | | | | |
| 2015 | 0.4132726 | 0.1275629 | −2.86 | 0.004 | 0.2256837 | 0.7567858 |
| Age (in months) | 1.105732 | 0.0231387 | 4.80 | 0.000 | 1.061298 | 1.152025 |
| Age quadratic | 0.9990266 | 0.0002739 | −3.55 | 0.000 | 0.99849 | 0.9995635 |
| Area | | | | | | |
| Peri-urban | 0.6929889 | 0.263014 | −0.97 | 0.334 | 0.3293556 | 1.458101 |
| Rural | 1.461653 | 0.4435081 | 1.25 | 0.211 | 0.8064269 | 2.649254 |
| Sex | | | | | | |
| Female | 0.9300564 | 0.1372602 | −0.49 | 0.623 | 0.6964448 | 1.24203 |
| Use of a bed net | | | | | | |
| No | 0.7451836 | 0.3585077 | −0.61 | 0.541 | 0.2902354 | 1.91327 |
| SES score | | | | | | |
| 2 | 0.8279565 | 0.2171552 | −0.72 | 0.472 | 0.4951715 | 1.384393 |
| 3 | 0.8945881 | 0.2246348 | −0.44 | 0.657 | 0.5468684 | 1.463401 |
| 4 | 0.8165903 | 0.2126572 | −0.78 | 0.437 | 0.4901551 | 1.360426 |
| Hygiene score | | | | | | |
| 1 | 1.179371 | 0.217316 | 0.90 | 0.371 | 0.8218725 | 1.692375 |
| 2 | 0.9610892 | 0.1934912 | −0.20 | 0.844 | 0.6477324 | 1.42604 |
| 3 | 0.5595068 | 0.1565725 | −2.08 | 0.038 | 0.3232994 | 0.968291 |
| Mother educated | 0.8735253 | 0.3427208 | −0.34 | 0.730 | 0.4048677 | 1.884681 |
| Polygamous HH | 0.8859926 | 0.1476902 | −0.73 | 0.468 | 0.6390567 | 1.228346 |
| Farming HH | 1.234606 | 0.3632742 | 0.72 | 0.474 | 0.6935344 | 2.197801 |
| HH with cattle | 2.208467 | 0.757137 | 2.31 | 0.021 | 1.127899 | 4.324257 |
| Distance to CHW | 1.114681 | 0.0411456 | 2.94 | 0.003 | 1.036885 | 1.198313 |
| Number of siblings | 1.113983 | 0.0460319 | 2.61 | 0.009 | 1.027319 | 1.207958 |
| _cons | 0.0024692 | 0.0015313 | −9.68 | 0.000 | 0.0007323 | 0.0083257 |
| Child | | | | | | |
| Var (_cons) | 0.9263946 | 0.1492716 | – | – | 0.6755235 | 1.270433 |

CHW = community health worker; CI = confidence interval; HH = household; RR = risk ratio; SES = socio-economic status.

SUPPLEMENTAL FILE 3: ANTI-PLACEBO TESTS ON SIMILARITY OF CHANGES BETWEEN EXPOSED AND NONEXPOSED GROUPS IN PRE-INTERVENTION PERIOD

**Outcome 1: point prevalence of malaria.**

Multilevel mixed-effects logistic model.

Outcome variable (rdt) equals 1 if parasite *lactate dehydrogenase*-based rapid diagnostic test (RDT) is positive.

The group variable (group) equals 1 if the child belongs to the group that will receive seasonal malaria chemoprevention in 2015.

The year (year) of reference is 2013.

The trend pre-intervention is expressed by the interaction term between the group variable and the year (group#year).

| rdt | Coefficient | Standard error | *z* | *P* > *z* | [95% CI] | |
|---|---|---|---|---|---|---|
| group | 0.1882329 | 0.2080876 | 0.90 | 0.366 | −0.2196113 | 0.596077 |
| year | | | | | | |
| 2014 | −0.3250146 | 0.2064955 | −1.57 | 0.115 | −0.7297384 | 0.0797092 |
| group#year | | | | | | |
| 1 2014 | −0.1459838 | 0.2611764 | −0.56 | 0.576 | −0.6578802 | 0.3659126 |
| Age (in months) | 0.0977595 | 0.018396 | 5.31 | 0.000 | 0.061704 | 0.1338149 |
| Area | | | | | | |
| Peri-urban | 0.3594561 | 0.3148713 | 1.14 | 0.254 | −0.2576803 | 0.9765925 |
| Rural | 1.000109 | 0.2795752 | 3.58 | 0.000 | 0.4521521 | 1.548067 |
| Age quadratic | −0.0009227 | 0.0002461 | −3.75 | 0.000 | −0.001405 | −0.0004404 |
| Sex | | | | | | |
| Female | −0.0233568 | 0.1396074 | −0.17 | 0.867 | −0.2969824 | 0.2502687 |
| Use of a bed net | | | | | | |
| No | 0.1371575 | 0.2496408 | 0.55 | 0.583 | −0.3521294 | 0.6264444 |
| SES score | | | | | | |
| 2 | −0.3898112 | 0.2520511 | −1.55 | 0.122 | −0.8838223 | 0.1041998 |
| 3 | −0.2015341 | 0.2355975 | −0.86 | 0.392 | −0.6632968 | 0.2602286 |
| 4 | −0.0931039 | 0.2349089 | −0.40 | 0.692 | −0.5535169 | 0.3673092 |
| Hygiene score | | | | | | |
| 1 | 0.0982546 | 0.1750381 | 0.56 | 0.575 | −0.2448138 | 0.4413229 |
| 2 | −0.2134621 | 0.2009579 | −1.06 | 0.288 | −0.6073323 | 0.1804081 |
| 3 | −0.1231137 | 0.2425915 | −0.51 | 0.612 | −0.5985844 | 0.3523569 |
| Mother educated | −0.0010933 | 0.3526535 | −0.00 | 0.998 | −0.6922815 | 0.690095 |
| Polygamous HH | −0.1301529 | 0.1657 | −0.79 | 0.432 | −0.4549189 | 0.1946131 |
| Farming HH | 0.3398964 | 0.2677388 | 1.27 | 0.204 | −0.1848621 | 0.8646548 |
| HH with cattle | 0.6600527 | 0.2981714 | 2.21 | 0.027 | 0.0756475 | 1.244458 |
| Distance to CHW | 0.1416596 | 0.0418179 | 3.39 | 0.001 | 0.0596981 | 0.2236212 |
| Number of siblings | 0.1768019 | 0.0395106 | 4.47 | 0.000 | 0.0993626 | 0.2542412 |
| _cons | −6.029974 | 0.6317766 | −9.54 | 0.000 | −7.268234 | −4.791715 |
| Child | | | | | | |
| Var (_cons) | 0.9393102 | 0.3415088 | – | – | 0.460611 | 1.915507 |

CHW = community health worker; CI = confidence interval.

**Outcome 2: period prevalence of malaria.**

Multilevel mixed-effects logistic model.

Outcome variable (rdt2) equals 1 if *Histidine-rich protein II*-based rapid diagnostic test (RDT) is positive.

The group variable (group) equals 1 if the child belongs to the group that will receive seasonal malaria chemoprevention in 2015.

The year (year) of reference is 2013.

The trend pre-intervention is expressed by the interaction term between the group variable and the year (group#year).

| rdt2 | Coefficient | Standard error | *z* | *P* > *z* | [95% CI] | |
|---|---|---|---|---|---|---|
| group | 0.0215083 | 0.192032 | 0.11 | 0.911 | −0.3548674 | 0.3978841 |
| year | | | | | | |
| 2014 | −0.7068914 | 0.1857018 | −3.81 | 0.000 | −1.07086 | −0.3429225 |
| group#year | | | | | | |
| 1 2014 | 0.2068579 | 0.2280316 | 0.91 | 0.364 | −0.2400759 | 0.6537917 |
| Age (in months) | 0.1180773 | 0.015059 | 7.84 | 0.000 | 0.0885622 | 0.1475923 |
| Area | | | | | | |
| Peri-urban | 0.2342814 | 0.2382207 | 0.98 | 0.325 | −0.2326226 | 0.7011853 |
| Rural | 1.12105 | 0.2183072 | 5.14 | 0.000 | 0.693176 | 1.548925 |
| Age quadratic | −0.0010118 | 0.0002033 | −4.98 | 0.000 | −0.0014102 | −0.0006133 |
| Sex | | | | | | |
| Female | 0.1279209 | 0.1272239 | 1.01 | 0.315 | −0.1214333 | 0.3772752 |
| Use of a bed net | | | | | | |
| No | −0.3138084 | 0.2328691 | −1.35 | 0.178 | −0.7702235 | 0.1426068 |
| SES score | | | | | | |
| 2 | −0.4001433 | 0.2164653 | −1.85 | 0.065 | −0.8244075 | 0.0241209 |
| 3 | −0.4502197 | 0.2087437 | −2.16 | 0.031 | −0.8593497 | −0.0410896 |
| 4 | −0.1411098 | 0.2103839 | −0.67 | 0.502 | −0.5534546 | 0.271235 |
| Hygiene score | | | | | | |
| 1 | −0.0639582 | 0.1660566 | −0.39 | 0.700 | −0.3894231 | 0.2615067 |
| 2 | −0.0654957 | 0.1811933 | −0.36 | 0.718 | −0.420628 | 0.2896366 |
| 3 | −0.4830406 | 0.2149736 | −2.25 | 0.025 | −0.9043812 | −0.0617 |
| Mother educated | −0.2067436 | 0.2912854 | −0.71 | 0.478 | −0.7776525 | 0.3641653 |
| Polygamous HH | −0.0392046 | 0.1496453 | −0.26 | 0.793 | −0.332504 | 0.2540948 |
| Farming HH | 0.0450637 | 0.2113421 | 0.21 | 0.831 | −0.3691593 | 0.4592866 |
| HH with cattle | 0.4229695 | 0.2195563 | 1.93 | 0.054 | −0.007353 | 0.853292 |
| Distance to CHW | 0.185835 | 0.0444062 | 4.18 | 0.000 | 0.0988005 | 0.2728696 |
| Number of siblings | 0.1513881 | 0.0385906 | 3.92 | 0.000 | 0.075752 | 0.2270243 |
| _cons | −3.817214 | 0.4791278 | −7.97 | 0.000 | −4.756288 | −2.878141 |
| Child | | | | | | |
| Var (_cons) | 1.397446 | 0.3406485 | – | – | 0.8666464 | 2.253348 |

CHW = community health worker; CI = confidence interval.

**Outcome 3: prevalence of moderate to severe anemia.**

Multilevel mixed-effects logistic model.

Outcome variable (anemic) equals 1 if the hemoglobin is less than 10 g/dL.

The group variable (group) equals 1 if the child belongs to the group that will receive seasonal malaria chemoprevention in 2015.

The year (year) of reference is 2013.

The trend pre-intervention is expressed by the interaction term between the group variable and the year (group#year).

| Anemic | Coefficient | Standard error | *z* | *P* > *z* | [95% CI] | |
|---|---|---|---|---|---|---|
| group | −0.0410647 | 0.163854 | −0.25 | 0.802 | −0.3622127 | 0.2800833 |
| year | | | | | | |
| 2014 | −0.2064313 | 0.1671188 | −1.24 | 0.217 | −0.5339781 | 0.1211155 |
| group#year | | | | | | |
| 1 2014 | 0.0644761 | 0.2074445 | 0.31 | 0.756 | −0.3421077 | 0.4710599 |
| Age (in months) | 0.0132072 | 0.0122248 | 1.08 | 0.280 | −0.010753 | 0.0371674 |
| Area | | | | | | |
| Peri-urban | 0.4659753 | 0.2037297 | 2.29 | 0.022 | 0.0666724 | 0.8652782 |
| Rural | 0.0033343 | 0.1834285 | 0.02 | 0.985 | −0.3561789 | 0.3628475 |
| Age quadratic | −0.0005915 | 0.0001815 | −3.26 | 0.001 | −0.0009472 | −0.0002359 |
| Sex | | | | | | |
| Female | −0.312014 | 0.1095497 | −2.85 | 0.004 | −0.5267275 | −0.0973004 |
| Use of a bed net | | | | | | |
| No | −0.1621162 | 0.2110839 | −0.77 | 0.442 | −0.575833 | 0.2516006 |
| SES score | | | | | | |
| 2 | 0.091807 | 0.1875675 | 0.49 | 0.625 | −0.2758185 | 0.4594325 |
| 3 | 0.2621982 | 0.1786185 | 1.47 | 0.142 | −0.0878877 | 0.6122841 |
| 4 | 0.2417127 | 0.1821366 | 1.33 | 0.184 | −0.1152685 | 0.5986939 |
| Hygiene score | | | | | | |
| 1 | 0.2314345 | 0.1418447 | 1.63 | 0.103 | −0.0465759 | 0.509445 |
| 2 | 0.1795424 | 0.1553966 | 1.16 | 0.248 | −0.1250294 | 0.4841141 |
| 3 | −0.2470947 | 0.1843121 | −1.34 | 0.180 | −0.6083398 | 0.1141504 |
| Mother educated | −0.0132136 | 0.2479968 | −0.05 | 0.958 | −0.4992784 | 0.4728512 |
| Polygamous HH | −0.0397988 | 0.1296762 | −0.31 | 0.759 | −0.2939595 | 0.2143619 |
| Farming HH | −0.1412905 | 0.1798477 | −0.79 | 0.432 | −0.4937856 | 0.2112046 |
| HH with cattle | 0.5136959 | 0.1934463 | 2.66 | 0.008 | 0.1345482 | 0.8928436 |
| Distance to CHW | 0.1017237 | 0.0354509 | 2.87 | 0.004 | 0.0322411 | 0.1712062 |
| Number of siblings | 0.0863406 | 0.0325824 | 2.65 | 0.008 | 0.0224802 | 0.150201 |
| _cons | −0.854311 | 0.3782572 | −2.26 | 0.024 | −1.595682 | −0.1129405 |
| Child | | | | | | |
| Var (_cons) | 0.6760422 | 0.2423205 | – | – | 0.3348646 | 1.364829 |

CHW = community health worker; CI = confidence interval.

**Outcome 4: history of fever.**

Multilevel mixed-effects logistic model.

Outcome variable (fever) equals 1 if a fever episode was reported during the 14 days before the survey.

The group variable (group) equals 1 if the child belongs to the group that will receive seasonal malaria chemoprevention in 2015.

The year (year) of reference is 2013.

The trend pre-intervention is expressed by the interaction term between the group variable and the year (group#year).

| fever | Coefficient | Standard error | *z* | *P* > *z* | [95% CI] | |
|---|---|---|---|---|---|---|
| group | 0.3503076 | 0.1722361 | 2.03 | 0.042 | 0.0127312 | 0.6878841 |
| year | | | | | | |
| 2014 | −0.0986139 | 0.1744483 | −0.57 | 0.572 | −0.4405262 | 0.2432985 |
| group#year | | | | | | |
| 1 2014 | −0.3450633 | 0.2179369 | −1.58 | 0.113 | −0.7722118 | 0.0820852 |
| Age (in months) | 0.0390556 | 0.0128352 | 3.04 | 0.002 | 0.0138992 | 0.0642121 |
| Area | | | | | | |
| Peri-urban | 0.2181614 | 0.2064828 | 1.06 | 0.291 | −0.1865374 | 0.6228602 |
| Rural | 0.1964259 | 0.1872495 | 1.05 | 0.294 | −0.1705763 | 0.563428 |
| Age quadratic | −0.0005593 | 0.0001834 | −3.05 | 0.002 | −0.0009187 | −0.0001998 |
| Sex | | | | | | |
| Female | −0.0919315 | 0.1106587 | −0.83 | 0.406 | −0.3088187 | 0.1249556 |
| Use of a bed net | | | | | | |
| No | −0.7582612 | 0.2474758 | −3.06 | 0.002 | −1.243305 | −0.2732177 |
| SES score | | | | | | |
| 2 | 0.1149772 | 0.1895516 | 0.61 | 0.544 | −0.2565372 | 0.4864916 |
| 3 | 0.146998 | 0.1813954 | 0.81 | 0.418 | −0.2085305 | 0.5025265 |
| 4 | 0.2111448 | 0.1849445 | 1.14 | 0.254 | −0.1513397 | 0.5736293 |
| Hygiene score | | | | | | |
| 1 | −0.0760066 | 0.1444342 | −0.53 | 0.599 | −0.3590924 | 0.2070792 |
| 2 | −0.1044463 | 0.1576658 | −0.66 | 0.508 | −0.4134657 | 0.204573 |
| 3 | −0.3171289 | 0.1883996 | −1.68 | 0.092 | −0.6863854 | 0.0521276 |
| Mother educated | −0.1602085 | 0.261492 | −0.61 | 0.540 | −0.6727234 | 0.3523064 |
| Polygamous HH | 0.2158298 | 0.1306106 | 1.65 | 0.098 | −0.0401623 | 0.4718219 |
| Farming HH | 0.044913 | 0.1825451 | 0.25 | 0.806 | −0.3128688 | 0.4026948 |
| HH with cattle | −0.0979718 | 0.1891979 | −0.52 | 0.605 | −0.4687929 | 0.2728493 |
| Distance to CHW | −0.0916116 | 0.0373225 | −2.45 | 0.014 | −0.1647623 | −0.0184609 |
| Number of siblings | −0.0333687 | 0.0333741 | −1.00 | 0.317 | −0.0987809 | 0.0320434 |
| _cons | −1.527117 | 0.3905396 | −3.91 | 0.000 | −2.29256 | −0.7616733 |
| Child | | | | | | |
| Var (_cons) | 0.4929859 | 0.2350039 | – | – | 0.1936739 | 1.254868 |

CHW = community health worker; CI = confidence interval.
